# Supplementary material for: WordCommentsAnalyzer: A windows software tool for qualitative research
Source: F1000Res. 2018 Sep 4;7:536. Originally published 2018 May 3. [Version 2] doi: 10.12688/f1000research.14819.2 (PMC6137411; doi:10.12688/f1000research.14819.2)
Supplement: Supplementary file 3 [file f1000research-7-17614-s0002.tgz › b2a79496-4ada-47c4-9682-a99ce867836d.zip!/Eight Iranian Health Journals/Journal of Knowledge and Health_20.docx]

© Journal of Knowledge and Health. This work is licensed under CC BY 4.0 (https://creativecommons.org/licenses/by/4.0/).

Volume & Issue: دوره 8، شماره 2: 1392

Title: ارتباط بین اجزاي تيپ بدني و سن رسیدن به اوج نمو (Peak High Velocity) در پسران 7-10 ساله ایرانی

http://knh.shmu.ac.ir/index.php/site/article/view/53

مقدمه: یک متغیر مهم جهت بررسی بالیدگی در دوران بلوغ، سن رسیدن به اوج سرعت نمو قد می‌باشد که مانند سایر شاخص‌های بالیدگی تحت تأثير متغیرهايی مانند وضعیت جسمانی و تیپ بدن فرد است. هدف مطالعه حاضر، بررسی ارتباط اجزاء تيپ بدني و سن رسیدن به اوج نمو قد مي‌باشد. مواد و روش‌ها: افراد تحت مطالعه 313 نفر از پسران سنین 7-10 ساله عضو پایگاه‌های تابستانی مراکز بسیج استان‌ها بودند. تيپ بدني به‌روش هیث-کارتر و سن رسيدن به اوج نمو (PHV) از فرمول ميروالد به‌دست آمد که با آزمون همبستگی پیرسون و رگرسیون چندگانه تحلیل شدند. نتایج: نتایج همبستگی پیرسون نشان داد كه سن PHV با اجزای اکتومورفی همبستگی معنادار مستقیم و با اجزای مزومورفی و اندومورفی همبستگی معنادار منفی دارد. همبستگی کلی سه جزء تيپ بدني با سن PHV؛ 50/0 بود که به‌طور‌کلی 25% واریانس سن PHV را تبيین می‌کند. نتیجه تحلیل رگرسیون نیز نشان داد که جزء اندومورفی و اکتومورفی نقش معناداری در پیش‌بینی سن PHV داشتند، اما جزء مزومورفی پیش‌بینی‌کننده معناداری نبود. نتیجه‌گیری: سهم جزء اندومورفی در پیش‌بینی سن PHV نسبت‌به دو جزء دیگر قوی‌تر بود، اما جزء مزومورفی پیش‌بینی‌کننده معناداری نبود. همچنین جزء اکتومورفی پس از اندومورفی؛ از بالاترین قدرت پیشگویی برخوردار بود. تحقیقات این حیطه پیشنهاد می‌کند، افرادی که جزء اکتومورفی بیشتری دارند دارای فرایند بالیدگی جسمانی کندتر بوده و پیش‌شرط‌های فیزیولوژیک لازم برای بلوغ در بدن آنها دیرتر فراهم می‌شود. بنابراین عملکرد ضعیف احتمالی کودکان اکتومورف در ورزش ممکن است به فاصله بیشتر آنها از سن PHV مرتبط باشد. از اين‌رو مربیان باید در انتخاب ورزشکاران نونهال نیم‌نگاهی به این موضوع داشته باشند.

Title: تأثير فعاليت بدني منظم در منزل بر كيفيت زندگي بيماران تحت جراحي بای‌پس عروق کرونر

http://knh.shmu.ac.ir/index.php/site/article/view/21

مقدمه: بیماری‌های مزمن؛ ازجمله بیماری‌های قلبی، اثرات نامطلوبي بر عملکرد فیزیکی، روانی، اجتماعی و به‌طوركلي كيفيت زندگي بیماران مبتلا دارد. این مطالعه با هدف تعیین تأثير فعاليت بدني منظم در منزل بر كيفيت زندگي بيماران تحت جراحي بای‌پس عروق کرونر انجام شده است. مواد و روش‌ها: این مطالعه مداخله‌اي بر روی بیمارانی که تحت جراحي باي‌پس عروق كرونر (CABG) قرار گرفته بودند، انجام شد. 58 بیمار از مركز آموزشي- درماني سيدالشهداي شهر اروميه به‌صورت تصادفی انتخاب شدند و سپس به‌روش تصادفی در دو گروه کنترل و مداخله قرار گرفتند. داده‌ها با استفاده از پرسشنامه کیفیت زندگی مک نیو جمع‌آوری گردید. بيماران گروه مداخله، حداقل هر نفر در 3 جلسه تحت آموزش ورزش‌های ترکیبی قرار گرفتند و اين ورزش‌ها را به‌مدت 12 هفته در منزل انجام دادند. تجزيه‌و‌تحليل داده‌ها با استفاده از آزمون‌هاي آماری كاي دو، من ويتني و ویلکاکسون انجام شده است. نتايج: یافته‌های مطالعه نشان‌دهنده ارتقاي کیفیت زندگی بیماران گروه مداخله در ابعاد جسمی، عاطفی و اجتماعی بعد از انجام فعالیت فیزیکی منظم در منزل نسبت‌به قبل از مداخله بود (001/0P<). همچنين ميانگين نمره کیفیت زندگی و كليه ابعاد آن در گروه مداخله به‌طور معناداري بيشتر از گروه كنترل بود (001/0P<). نتیجه‌گیری: انجام فعالیت بدني منظم در منزل اثرات مهمی بر روی ارتقاي کیفیت زندگی بیماران بعد از جراحي بای‌پس عروق کرونر دارد، لذا پيشنهاد مي‌شود بیماران بعد از CABG در مورد انجام فعالیت فیزیکی منظم در منزل آموزش ديده و به انجام آن تشويق شوند.

Title: تأثير فعالیت مقاومتی حاد بر فشارخون و ادراک درد بیماران دیابتی نوع ۲ دارای اضافه‌وزن

http://knh.shmu.ac.ir/index.php/site/article/view/25

مقدمه: تعداد بیماران دیابتی در دهه اخیر افزایش فزاینده‌ای داشته است و درد به‌عنوان یکی از عمده‌ترین ناراحتی‌های این بیماران مطرح شده است. از‌سوی‌دیگر، در مورد اثر فعالیت مقاومتی بر کاهش درد اطلاعات کافی وجود ندارد. بر این اساس، هدف از این پژوهش بررسی اثر فعالیت مقاومتی بر آستانه درد و فشارخون بیماران مبتلا به دیابت مي‌باشد. مواد و روش‌ها: 11 بیمار دیابتی (45-50 ساله) دارای اضافه‌وزن به‌صورت تصادفی هدفدار انتخاب شدند، اما 3 نفر از آنها از ادامه فعالیت خودداری کردند. پس از اندازه‌گیری یک تکرار بیشینه، فعالیت مقاومتی را ۳ نوبت با شدت ۸۰% یک تکرار بیشینه اجرا کردند. آستانه درد و فشارخون قبل و بعد از فعالیت از‌طریق دستگاه‌های ویژه در دست فعال اندازه‌گیری شد و درنهایت داده‌ها با استفاده از آزمون آماری t زوجي و ضریب همبستگی تجزیه‌و‌تحلیل شدند. نتايج: ميانگين فشارخون، فشارخون دياستولي، سيستولي و آستانه دارد و به‌طور معناداري بلافاصله پس از فعاليت مقاومتي نسبت‌به قبل از فعاليت افزايش يافته است (05/0P<). همچنين در اين مطالعه رابطه بین افزایش فشارخون دیاستولی و آستانه درد معنادار نبود. نتیجه‌گیری: براساس عمده یافته‌های این پژوهش، تقابل فشارخون و آستانه درد مشاهده شد که می‌تواند حاکی از مکانیزم‌های مرکزی ضد درد در افراد دیابتی باشد.

Title: مقايسه تزريق داخل ضايعه گلوكانتيم به تنهايي و توأماً با كرايوتراپي در درمان ليشمانيوز جلدي روستايي:كارآزمايي باليني تصادفي

http://knh.shmu.ac.ir/index.php/site/article/view/26

مقدمه: اين مطالعه با هدف مقایسه اثرات درمانی دو روش درماني تزریق داخل ضایعه گلوکانتیم و روش کرایوتراپی به همراه تزريق داخل ضايعه در درمان لیشمانیوز جلدي در استان قم انجام شده است. مواد و روش‌ها: در اين مطالعه كارآزمايي باليني، بيماران مبتلا به ليشمانيوز جلدي به‌صورت تصادفي به دو گروه اختصاص داده شدند. گروه اول تحت درمان با گلوکانتیم به ‌روش تزریق داخل ضایعه به‌طور هفتگی و گروه دوم تحت درمان با روش درماني توأماً تزريق داخل ضايعه و کرایوتراپی به‌صورت يك‌هفته در ميان قرار گرفتند. پس از 7 و 12 هفته، درمان ضایعات مورد پایش قرار ‌گرفت. بهبودي به‌صورت راپيتليازاسيون كامل و ازبين‌رفتن سفتي در نظر گرفته شد. نتايج: از 197 بيمار مبتلا ‌به سالك، 112 بیمار با عامل ليشمانيو ماژور، در مطالعه شرکت کرده و دوره درمان را کامل نمودند. 54 بیمار با 116 ضایعه، تزریق داخل ضایعه گلوکانتیم و 58 بیمار با 115 ضایعه، کرایوتراپی و تزریق داخل ضایعه به‌صورت توأماً را دريافت داشتند. در گروه گلوکانتیم با تزريق داخل ضايعه 1/48% ضایعات و در گروه کرایوتراپی به همراه تزريق داخل ضايعه 2/72% ضایعات پس از 7 نوبت درمان شدند و پس از 12 نوبت درمان، 100% ضايعات تحت درمان توأم و 91% ضايعات تحت درمان با گلوكانتيم داخل ضايعه از‌نظر باليني، بهبود كامل يافتند. برای پایان هفته هفتم خطر نسبی (حدود اطمینان) 86/1 (15/3 - 1/1) به‌دست‌آمد و برای پایان هفته دوازدهم 23/2 RR= و (3/4 - 7/2=CI95/0) محاسبه گرديد که از‌نظر آماری معنادار می‌باشد (02/0P<). بين ميزان بهبودي در دو گروه تحت درمان ازنظر آماري رابطه معناداري ديده شد (05/0P<). نتیجه‌گیری: کرایوتراپی به همراه تزريق داخل ضايعه به‌صورت توأماً در مقایسه با روش تزریق داخل ضایعه گلوکانتیم روش درماني مؤثرتري بوده و دوره درمان را كاهش مي‌دهد.

Title: بررسی تأثير آوای قرآن و موسیقی بدون کلام بر اضطراب بیماران کاندید آندوسکوپی

http://knh.shmu.ac.ir/index.php/site/article/view/28

مقدمه: صدا درمانی، یکی از روش‌های درمانی طب مکمل بوده که در بسیاری از بیماری‌ها استفاده قرار می‌شود. با‌توجه ‌به اینکه سطح اضطراب در بیماران کاندید آندوسکوپی بالا می‌باشد، هدف از مطالعه حاضر، بررسی تأثير آوای قرآن و موسیقی بدون کلام بر میزان اضطراب بیماران کاندید آندوسکوپی می‌باشد. مواد و روش‌ها: این مطالعه یک كارآزمايي باليني بوده که برروی 60 نفر از بیماران مراجعه‌کننده به یک مطب خصوصی بیماری‌های گوارش انجام گردیده است. بیماران به‌طور تصادفی به سه گروه آوای قرآن، موسیقی و شاهد تقسیم شدند. اطلاعات با استفاده از دو پرسشنامه اطلاعات دموگرافیک و پرسشنامه استاندارد اسپیل برگر، برای سنجش اضطراب جمع‌آوری گردید. نتایج: نتایج حاصل از این مطالعه نشان داد كه ميانگين اضطراب در سه گروه، بعد از مداخله تفاوت معناداري دارند و مقايسه نمرات اضطراب قبل و بعد از مداخله نشان داد كه استماع قرآن (001/0=P) و موسيقي (03/0=P) نسبت‌به عدم‌مداخله (09/0=P) بر سطح اضطراب بيماران تأثير مثبت دارد. در گروه استماع آوای قرآن، ميانگين اضطراب کمتری نسبت‌به ساير گروه‌ها گزارش شد. نتیجه‌گیری: با‌توجه‌ به اینکه آوای قرآن نسبت‌به موسیقی بدون کلام تأثير بیشتری بر کاهش اضطراب بیماران داشته است، استفاده از آوای قرآن قبل از انجام کلیه اقدامات درمانی همراه با سطح اضطراب بالا توصیه می‌گردد.

Title: مقایسه کیفیت زندگی بیماران مبتلا به دیابت با بیماران مبتلا به سل

http://knh.shmu.ac.ir/index.php/site/article/view/30

مقدمه: دیابت و سل از شایع‌ترین بیماری‌های عصر حاضر است که با اثر بر ابعاد مختلف زندگی، کیفیت زندگی بیماران را تحت تأثير قرار می‌دهد. ازآنجایي‌كه‌‌‌‌‌‌‌ کیفیت زندگی برآیند اصلی اقدامات درمانی و مراقبتی است، مطالعه حاضر به‌منظور مقایسه و مشخص‌ترنمودن اثرات متفاوت بیماری دیابت و بیماری سل بر روی کیفیت زندگی صورت گرفته است. مواد و روش‌ها: پژوهش حاضر یک مطالعه توصیفی- تحلیلی است که برروی 320 بیمار مبتلا به دیابت و سل مراجعه‌کننده به چهار درمانگاه دیابت و درمانگاه مرکز ارجاع سل در شهر تهران انجام شده است. به‌منظور جمع‌آوري اطلاعات، از پرسشنامه مشخصات فردی و نسخه فارسي فرم کوتاه 36 استفاده گرديد. نتایج: در این پژوهش‌ 131 بیمار مبتلا به دیابت و 189بيمار مبتلا به سل شرکت کردند. میانگین سنی بیماران مبتلا به دیابت 7/12 ±3/47 سال و بیماران مبتلا به سل 6/17 ±8/41 سال بوده است. دامنه نمرات کسب‌شده براي ابعاد مختلف کيفيت زندگي بیماران مبتلا به دیابت از 9/12±2/46 براي سلامت عمومي تا 61/26±13/64 براي عملکرد جسمي متغير بوده است. دامنه نمرات کسب‌شده براي ابعاد مختلف كيفيت زندگي بیماران مبتلا به سل از 58/32± 85/16 براي ايفاي نقش جسمي تا 55/22±18/59 براي عملکرد اجتماعي متغير بوده است. نتایج حاصل از تجزیه‌وتحلیل آماری نشان داد که بیماران مبتلا به سل به‌طور معناداری نمرات پایین‌تری در ابعاد عملکرد جسمی، ایفای نقش جسمی، انرژی و نشاط، دردهای بدنی و ایفای نقش عاطفی از بیماران مبتلا به دیابت کسب کردند. بیماران مبتلا به دیابت به‌طور معناداری نمره پایین‌تری در بعد سلامت عمومی از بیماران مبتلا به سل کسب کردند. نتیجه‌گیری: بیماری‌های دیابت و سل اثرات متفاوتی بر روی کیفیت زندگی دارند. توجه به این تفاوت‌ها و عوامل اثرگذار به‌منظور استفاده در برنامه‌های درمانی- مراقبتی ارتقاي کیفیت زندگی این بیماران ضروری است.

Title: بررسی میزان شیوع دردهای عضلاني- اسكلتي و ارتباط آن با ناتوانی عملکردی در سالمندان

http://knh.shmu.ac.ir/index.php/site/article/view/50

مقدمه: در فرایند سالمندی، سیستم‌های مختلف بدن تحلیل رفته و باعث افزایش وابستگی سالمندان به دیگران مي‌شود. هدف از این مطالعه، بررسی میزان شیوع دردهای عضلانی- اسکلتی و ارتباط آن با ناتوانی عملکردی در سالمندان مي‌باشد. مواد و روش‌ها: در این مطالعه مقطعی، 1614 سالمند شهر امیرکلا در سال 90-91 شرکت داشتند. داده‌ها با استفاده از پرسشنامه‌های اطلاعات دموگرافیک ناتوانی عملکردی درد در سالمندان جمع‌آوری شد. رابطه بين درد و ناتواني عملكردي با استفاده از آزمون كاي- دو در سطح 05/0 بررسي شد. نتایج: در این مطالعه 7/54% مرد و 3/45% زن بودند. درد در 1330 نفر (4/82%) گزارش شد که 5/47% در اندام فوقانی، 2/73% در اندام تحتانی و 5/24% در سر و گردن احساس درد مي‌کردند. شایع‌ترین محل درد اندام فوقانی در شانه، 606 نفر (5/37%) و اندام تحتانی در زانو، 1018 نفر (1/63%) گزارش شد. درد در زنان بیشتر از مردان دیده شد و در مردان و زنان در اندام تحتانی (زانو) بیشتر از سایر نقاط بدن درد مشاهده شد. در این بررسی 7/3% از افراد دچار ناتوانی عملکردی بودند. ارتباط معناداری بین درد و ناتوانی عملکردی وجود نداشت (24/0P=). نتیجه‌گیری: نتایج این مطالعه نشان‌دهنده شیوع بالای درد عضلانی اسکلتی در سالمندان شهر امیرکلا مي‌باشد. شیوع درد در زنان بیشتر از مردان بوده و شایع‌ترین محل درد ناحیه زانو گزارش شده است كه نيازمند مداخلات درماني، به‌خصوص آموزش لازم جهت پيشگيري مي‌باشند.

Title: بررسي پیشگویی‌کننده‌های رفتار پیشگیری از مواجهه با آلودگی هوا در زنان باردار:یک مطالعه مبتني بر الگوی فرانظریه‌ای

http://knh.shmu.ac.ir/index.php/site/article/view/52

مقدمه: باتوجه به مشكلات مادري و جنيني مرتبط با مواجهه زنان باردار با آلودگي هوا و نقش تعيين مراحل تغيير رفتار در پيشگيري از مواجهه، مطالعه حاضر با هدف تعیین پیشگویی‌کننده‌های مراحل تغییر رفتاری پیشگیری از مواجهه با آلودگی هوا در زنان باردار شهر تهران طراحی شده است. مواد و روش‌ها: در یک مطالعه مقطعی 200 نمونه از زنان باردار سه منطقه جغرافیایی شمال، جنوب و مرکز تهران که به درمانگاه‌های مراقبت دوران بارداری مراجعه کردند، به‌طور تصادفی وارد مطالعه شدند. جمع‌آوری داده‌ها ازطریق پرسشنامه‌های محقق ساخته و استانداردشده مطابق با الگوی فرانظریه‌ای، شامل فرم اطلاعات مردم‌شناختی، پرسشنامه منافع، موانع، خودکارآمدی و مراحل تغییر انجام پذیرفت. تجزیه‌و‌تحلیل داده‌ها با استفاده از آزمون كای- دو، ضریب همبستگی اسپیرمن و رگرسیون لجستیک انجام شد. نتایج: میانگین (±انحراف معیار) سني افراد مورد مطالعه 4/8 ±9/26 سال و میانگین (±انحراف معیار) سن بارداری 1/9±9/27 هفته بود. بین مراحل تغییر رفتار با موانع، منافع و خودکارآمدی، همبستگی خطی معناداری مشاهده شد (001/0P=). همچنین یافته‌های پژوهش نشان داد که خودکارآمدی توان پیشگویی‌کنندگی مراحل تغییر رفتار را دارد (001/0P=). نتیجه‌گیری: نتایج این مطالعه نشان داد كه از ميان سازه‌هاي مورد بررسي الگوي فرانظریه‌ای، خودكارآمدي تنها سازه پيشگويي‌كننده اتخاذ رفتار پيشگيري از آلودگي هوا مي‌باشد. یافته‌های این پژوهش می‌تواند به‌عنوان پایه‌ای برای مداخلات آموزشی درزمینه تغییر رفتار جهت کاهش مواجهه با آلودگی هوا در زنان باردار، مورد استفاده متخصصان امر سلامت قرار گیرد.
